# Chronic Non-Exertional Myalgia and Myoadenylate Deaminase Deficiency: a Possible Association

**DOI:** 10.4021/jocmr2009.04.1231

**Published:** 2009-03-24

**Authors:** Aiesha Ahmed, Max R Lowden

**Affiliations:** a Penn State College of Medicine, Hershey Medical Center, Department of Neurology - EC037, 30 Hope Drive, Hershey, PA 17033, USA

## Abstract

Myoadenylate deaminase deficiency is noted in skeletal muscles. It generally presents with exertional myalgias, fatigue and weakness. We present a patient who complained of constant pain unrelated to activity with biopsy finding consistent of myoadenylate deaminase deficiency.

**Keywords:**

Myoadenylate deaminase deficiency; Myalgia; Pain

## Introduction

Myoadenylate deaminase catalyses the deamination of adenosine monophosphate to inosine monophosphate to release energy during muscle activity [1]. Myoadenylate deaminase deficiency is noted in skeletal muscles of 2-3% of patients with neuromuscular disorders [2]. Different isoforms are present in skeletal and smooth muscles, nerve bundles as well as in endothelial cells [3]. Clinical presentation is usually exertional myalgia and fatigue. We report a patient with biopsy proven myoadenylate deaminase deficiency which did not present with the usual exertion related muscle pain.

## Case Report

A 46-year-old white man presented to the neuromuscular clinic with generalized muscle pain, fatigue and cramps for 10-15 years. The muscle pain was described as constant, dull and achy. It was present during rest and was unchanged by exercise. There were no paresthesias, fever, swollen joints, rashes, or dark urine but the patient did admit to generalized weakness.

The patient had a past medical history of spontaneous pneumothoraces and hypercholesterolemia. Family history reveals no neuromuscular diseases. Interventions including physical therapy, aquatherapy, tizanidine, tricyclic antidepressants, and nonsteroidal anti-inflammatory drugs provided minimal relief.

On physical examination, he had intact muscle bulk, strength and tone throughout. His muscle stretch reflexes were normal. The remainder of the neurological examination was normal. Evaluations including blood work for connective tissue disorders, chemistry, thyroid studies, creatine phosphokinase and electrodiagnostic studies which failed to identify an etiology for his complaints.

**Figure 1:**
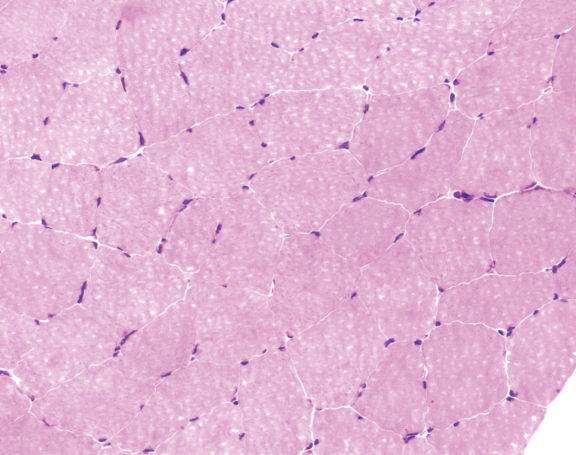
Hematoxylin and eosin stain of muscle showing mild variation in myofiber size and shape but overall preservation of normal morphology as typically seen in myoadenylate deaminase deficiency. x 400.

**Figure 2:**
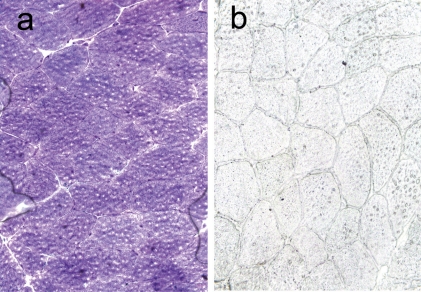
Adenosine monophosphate deaminase stain of muscle showing normal control (a) and markedly diminished myoadenylate deaminase activity (b). x 400.

A muscle biopsy of the left vastus lateralis was performed in light of persistent symptomatology. It showed mild morphologic abnormalities consistent with variation in myofiber size and shape (Fig. 1). Myofiber glycogen content and staining for phosphorylase, phosphofructokinase, and acid phosphatase were unrevealing. Staining for myoadenylate deaminase activity was markedly diminished (Fig. 2). Biochemical analyses for acid maltase, fatty acid transport (carnitine, carnitine palmitoyltransferase) and mitochondrial enzymes including respiratory chain enzymes were unremarkable.

## Discussion

Exertional myalgia is seen with myoadenylate deaminase deficiency in most of the reported cases [4-7], along with fatigue and weakness. We present a case of longstanding constant pain which was not triggered by exertion. We found a few reports of resting pain in patients with myoadenylate deaminase deficiency, however these patients developed it either subsequent to having exertional mylagia for some time [3] or the pain was minimal at rest and would worsen on exertion [8].

In summary, our patient's presentation of constant mylagias in the absence of laboratory work up suggesting a neuromuscular disorder other than biopsy proven myoadenylate deaminase deficiency is unusual. Investigations for connective tissue disorder, infections and malignancy were also unremarkable. We approached muscle biopsy as a last step to exclude all possibilities. The biopsy results showing remarkable absence of myoadenylate deaminase bring ups the question that this deficiency may be associated with symptoms of constant pain. We suggest that chronic constant myalgias should be considered as a possible uncommon presentation for myoadenylate deaminase deficiency and good clinical judgment and exclusion of other etiologies should be undertaken.
